# The intergenerational effects of low parental socio‐economic position on cardiometabolic and inflammatory outcomes: A systematic review and meta‐analysis

**DOI:** 10.1111/eci.70125

**Published:** 2025-09-29

**Authors:** Juan Carlos Rivillas‐García, Emilie Courtin, Eleanor Winpenny, Olaide Adebayo‐Clement, Raúl Devia‐Rodríguez, Ornella Moreno‐Mattar, Paolo Vineis

**Affiliations:** ^1^ Department of Epidemiology and Biostatistics, School of Public Health Imperial College London London UK; ^2^ Department of Health Policy London School of Economics and Political Sciences (LSE) London UK; ^3^ School of Public Health Imperial College London London UK; ^4^ MRC Environment and Health, School of Public Health Imperial College London London UK; ^5^ University Medical Center Groningen (UMCG), University of Groningen Groningen The Netherlands; ^6^ Health Inequalities Research Group/Employment Conditions Network (GREDS‐EMCO NET), JHU‐UPF Public Policy Center (JHU‐UPF PPC) Universitat Pompeu Fabra (UPF), UPF Barcelona, School of Management (UPF‐BSM) Barcelona Spain; ^7^ MRC Centre for Environment and Health, School of Public Health Imperial College London London UK

**Keywords:** biological mechanisms, cardiometabolic biomarkers, inflammation, life course epidemiology, parental socio‐economic position

## Abstract

**Background:**

Evidence on the impacts of parental and early life socio‐economic position (SEP) on health outcomes in adulthood remains mixed. This systematic review and meta‐analysis investigated the association between low parental SEP and adult cardiometabolic and inflammatory markers in individuals aged 18 years and older.

**Methods:**

A systematic search across five databases (EMBASE, Ovid MEDLINE, Cinahl, Global Health and Maternity and Infant Care until January 01, 2022) identified observational studies linking parental SEP with adult cardiometabolic and inflammatory markers. Pooled Standardized Mean Differences (SMD) were estimated using random‐effects models. Risk of bias, heterogeneity and publication bias were assessed using the Cochrane tool, subgroup analysis and Egger's test, respectively.

**Results:**

The review included 38 studies (12 in meta‐analysis, *n* = 388,674). Findings showed that lower parental SEP was significantly associated with elevated blood pressure (SMD = .30 mmHg; 95% CI: .10, .50; *I* ^2^ 94%; *n* = 5), increased adiposity (SMD = .56; 95% CI: .05, 1.07: *I* ^2^ 98%; *n* = 6), higher C‐reactive protein levels (SMD = 1.45 mg/dL; 95% CI: .06, 2.85; *I* ^2^ 80%; *n* = 9), elevated IL‐6 (SMD = 2.12 pg./mL; 95% CI: −.72, 4.97; *I* ^2^ 100%; *n* = 4) and higher allostatic load (SMD = .85; 95% CI: .30, 1.40; *I* ^2^ 99%; *n* = 4). No consistent associations were found for glucose or lipid markers. Gender‐specific variations were observed.

**Conclusions:**

Low parental socio‐economic position negatively impacts adult offspring health, manifesting as higher blood pressure, elevated C‐reactive protein, increased interleukin‐6, greater adiposity and higher allostatic load. Future research should prioritise three critical areas: mechanistic specificity, intersectional pathways and life‐course timing and critical period detection.

## Graphical abstract

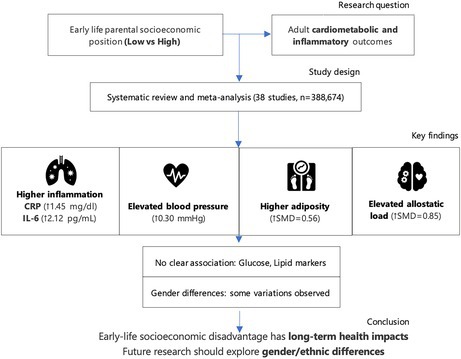

## 1 INTRODUCTION

Socio‐economic position (SEP) incorporates multiple aspects such as access to resources, occupation, education and perceived social status.^1^, ^2^, ^3^ Early life adversity and psychosocial stress, including low childhood SEP, can alter biological functioning across the life course, increasing the risk of chronic disease^4^, ^5^, ^6^, ^7^, ^8^ and cognitive decline.^9^, ^10^, ^11^ Parental SEP captures various dimensions of early life adversities. Parental education reflects cognitive skills, knowledge and cultural capital, while parental occupation indicates job titles, social status and access to resources and opportunities.^7^, ^12^, ^13^, ^14^

Intergenerational health inequalities are a fundamental area of research in life course, social and ageing epidemiology. Investigating the impact of low parental SEP on later‐life disease can address unsolved key questions about determinants of health and longevity. While some studies suggest higher parental education and occupation protect against cardiometabolic and inflammatory dysregulation risk in children,^15^, ^16^ others have failed to confirm this.^17^, ^18^, ^19^ Associations between parental SEP and adult biological dysregulation also remain unclear in some research.^20^, ^21^, ^22^, ^23^, ^24^ Miller et al.^23^ examined how early‐life stress linked to low SEP may contribute to inflammation and metabolic risk in adulthood but noted variability in findings, while Cohen et al.^20^ found mixed evidence on parental SEP and adult biological markers, highlighting gaps in understanding mechanisms. Both studies acknowledged the complexity of SEP‐biology links.

We need a better understanding of all the different aspects of SEP and their impacts. A systematic review and meta‐analysis could help clarify these differences. While previous systematic reviews and meta‐analyses have explored the impact of parental SEP on biological dysregulation,^5^, ^6^, ^25^, ^26^ this review is the first attempt to comprehensively analyse the relationship between parental SEP and both cardiometabolic and inflammatory markers in adulthood, also addressing the limitations of prior research.

## 2 MATERIALS AND METHODS

### 2.1 Search strategy and selection criteria

A search of published studies was conducted in the following electronic databases from 1980 until January 2022: EMBASE, Ovid MEDLINE, Cinahl, Global Health and Maternity and Infant Care. The choice of the keywords was based on previously published reports^2^, ^22^ and adapted to each database. Table S1 describes the biomarkers used, and Appendix S1 describes keywords and search strategy. Hand‐searched reference lists and citations of included studies were also completed to identify additional relevant studies. Two reviewers (RDR and OMM) independently screened the retrieved reference lists for each database, abstract screening, removed duplicates and assessed the full text for eligibility using Covidence. Both reviewers searched for the results and compared the number of retrieved studies. A third reviewer addressed any disagreements regarding eligibility (JCR).

### 2.2 Inclusion and exclusion criteria

Criteria for inclusion were defined as below: (1) papers were full peer‐reviewed journal articles, (2) available in English or Spanish, (3) observational studies (longitudinal and cross‐sectional studies) conducted in adult participants (≥18 years), providing data on the association between parental SEP measures and adult cardiometabolic and inflammatory outcomes and (4) reporting effect estimates for the outcomes in the form of Standardised Mean Difference (SMD) and 95% confidence intervals (CIs). Exclusion criteria were described as follows: (1) Studies focusing on specific socio‐economic factors (e.g. employment, health insurance and area of residence); (2) covering other physiological systems or markers; and (3) studies undertaken in children and adolescents (<18 years).

### 2.3 Data extraction

Two researchers independently extracted information from 38 selected studies and coded individual studies using a customised extraction form (JCR and OAC). The form was piloted on five studies before reviewers proceeded to the complete data extraction. Data extracted from each article included: (1) study design (longitudinal or cross‐sectional, period of data collection, follow‐up and year of publication); (2) sample characteristics (country, number of men and women, age, ethnicity, sample size and number of individuals); (3) the used parental SEP measure (the means and standard deviation of the parental SEP groups); (4) the method of assessing parental SEP exposures (self‐reported questionnaire, clinical interview and record review); and (5) whether there were associations between exposures and cardiometabolic or inflammatory outcomes (e.g. odds ratios, mean differences between groups).

The effect size extracted from the articles and the test used to compute it were recorded by two reviewers. For studies that examined multiple exposures and health outcomes, data for each exposure and outcome within each group were extracted. Discrepancies between reviewers were discussed until a consensus was reached. Missing data were requested from the principal study's author by e‐mail. If we had yet to receive a positive response from the study's author, follow‐up emails were sent after 2 weeks. Inclusion of multiple estimates per study followed a priori criteria: (a) distinct outcomes (for instance, SBP and DBP if analysing blood pressure broadly or CRP and IL‐6 is studying inflammation); (b) independent subgroups (for instance, estimates for men vs. women if gender differences are a focus, or age‐stratified estimates); and (c) adjusted models prioritised to minimise confounding. This approach aligns with meta‐analytic guidelines while mitigating nonindependency bias.^27^, ^28^

### 2.4 Quality assessment

To assess the risk of bias in each study, the Cochrane Collaboration's Risk of Bias tool^29^ was used in the following domains: (1) eligibility criteria, (2) collection of exposures, (3) adjustments for potential confounders, (4) missing outcome data, (5) measurement of the outcome data and (6) selection of reported results. Studies with a high risk of bias in one domain were classified as having an increased risk of bias overall. Two reviewers independently assessed the risk of bias (JCR and OAC).

### 2.5 Statistical power

This review ended up by including a small number of studies for some specific markers, ranging from as little as two to a maximum of four studies, mainly cross‐sectional. To increase statistical power, we used two approaches, collapsing the main SEP measures and markers categories:

(1) Combining five parental SEP measures into a single parental SEP measure (low and high parental SEP). We decided on this approach as the mother's education, father's occupation, parental education, parental occupation and family SEP measures are similar operationalisations of the same construct (Table S1 describes the operationalisation of five parental SEP measures).

(2) Collapsing markers supported by clinical relevance and handling multiple outcomes from the same study^27^: (a) blood pressure included systolic blood pressure (SBP) and diastolic blood pressure (DBP) stratifying by study design (cohort vs. cross‐sectional). SBP and DBP can be analysed as related outcomes in the meta‐analysis^27^, ^28^; (b) metabolic markers: HbA1c, fasting glucose and blood glucose are biomarkers clinically interchangeable and were included in a single glucose metabolism group; (c) lipid metabolism outcomes included total cholesterol, triglyceride and low‐density lipoprotein (LDL). (d) Adiposity in cohort studies: body‐mass index and waist circumference were pooled to align with WHO metabolic syndrome criteria.^30^ Although BMI and WC capture different aspects of the body fat distribution (general vs. abdominal), both are established predictors of cardiometabolic risk.^31^ (e) inflammation outcomes included C‐reactive protein (CRP) and interleukin 6 (IL‐6). (f) Allostatic load was analysed separately only for the cross‐sectional studies. After collapsing into main groups of outcomes, only glucose metabolic and lipid metabolic markers still showed a small number of studies in the meta‐analysis (*n* = 4 studies).

### 2.6 Meta‐analysis

Results from selected studies were summarised based on exposures, outcomes and research designs, with characteristics detailed in Table S2. A random‐effects meta‐analysis was performed to calculate the standardized mean difference (SMD) and associated 95% confidence intervals (CIs), with pooled estimates reported using the metapackage in R.^27^, ^32^ Heterogeneity was assessed using Q and *I* ^2^ statistics with values of <25%, 25%–50%, 50%–75% and >75% interpreted as low, moderate, high and extreme heterogeneity, respectively.^33^ Meta‐analyses were conducted separately for cardiovascular, metabolic and inflammatory outcomes and for each study design. Forest plots illustrated effect sizes with 95% CIs for individual studies. Funnel plots were generated to visually assess publication bias.^33^, ^34^, ^35^ The meta package in R Studio^32^, ^36^ was used to assess statistical power by estimating the range of reliably detectable effect sizes at the individual study level.^37^ Codes and detailed tables with the number of observations, means and standard deviations are available in the Appendix S1 and on GitHub.

### 2.7 Synthesis of findings

Statistical analyses were performed by using R Studio version 4.1.2 (The R Foundation for Statistical Computing, Vienna, Austria). The standardised mean differences were estimated as overall effect sizes and reported as pooled SMD with 95% CI in random effects meta‐analyses.

Furthermore, statistical power was also assessed to determine the range of reliably detectable effect sizes and employing the ‘*metameta*’ R package. Detailed procedures, including codes and tables with the number of observations, means and standard deviations among exposed and nonexposed groups, are available in Appendix S1. Findings from studies not eligible for meta‐analysis, for instance due to incompatible effect sizes reporting or heterogeneous outcomes were synthesised narratively.

## 3 RESULTS

### 3.1 Study characteristics

**FIGURE 1:**
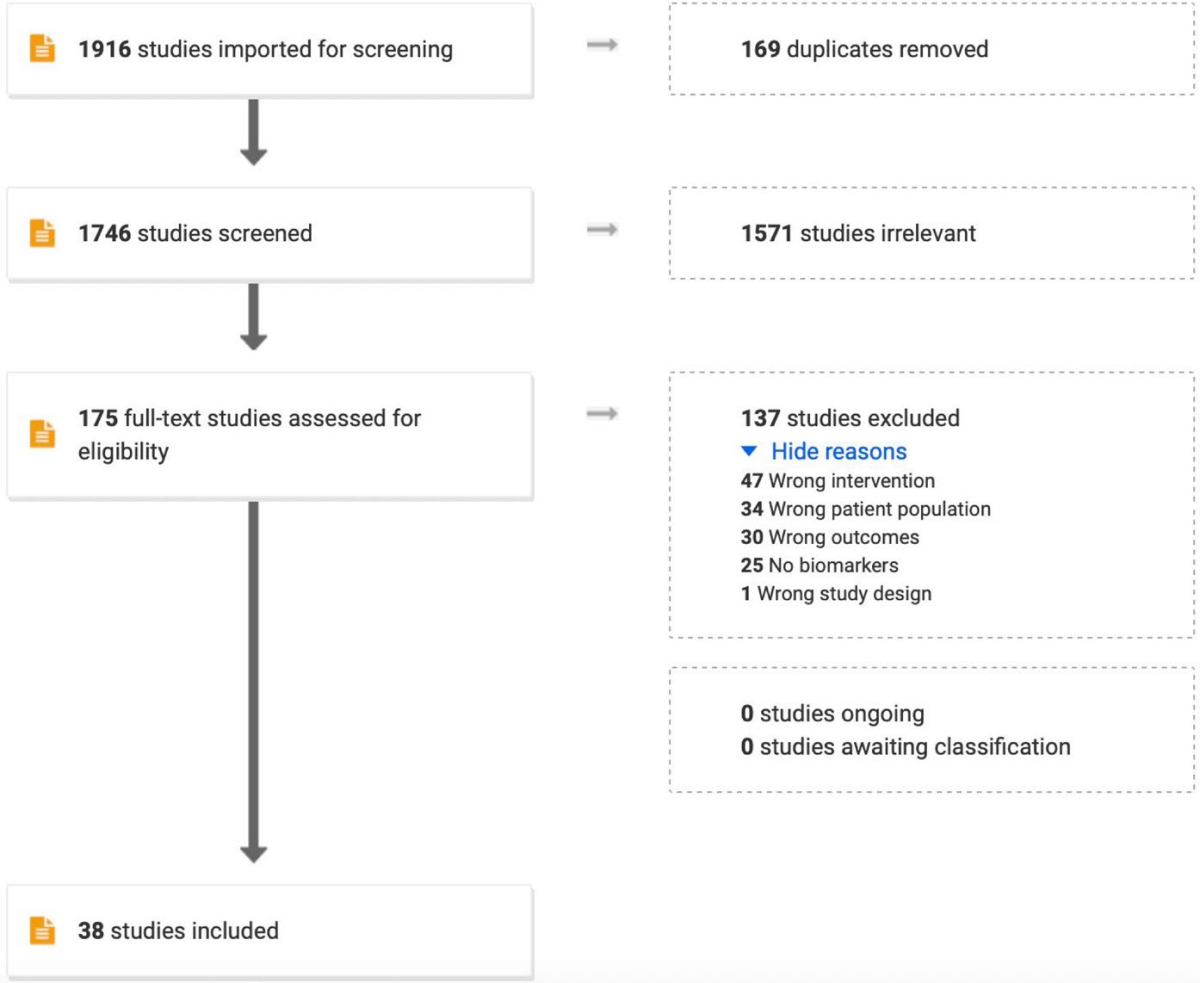
PRISMA flow diagram of study population selection process and profile.

Our systematic search identified 1916 studies, with 38 studies meeting inclusion criteria after screening (Figure 1). The excluded studies (*n* = 1878) primarily lacked relevant exposures, focused on paediatric populations or reported incompatible outcomes.

The analytical sample comprised 28 cohort studies (follow‐up range 1–60 years) and 10 cross‐sectional studies published between 1982 and 2022. Geographically, studies originated from Europe (*n* = 19), the Americas (*n* = 14), Asia (*n* = 2 studies) and one multinational cohort (Table 1). While 20 studies examined exclusively White populations, 16 reported stratified data by race/ethnicity, including six with specific ethnic groups' analysis.

**TABLE 1:** Summary characteristics of included studies.

Characteristics	Number of studies (*n* = 38)	Percentage of the studies %
Study design		
Cohort studies	28	74%
Cross‐sectional studies	10	26%
Location of the study population		
USA	13	34.2%
UK	7	18.4%
Finland	5	13.1%
Denmark	4	10.5%
Taiwan	2	5.3%
Multi‐cohort Europe	2	5.3%
Jamaica	1	2.6%
Sweden	1	2.6%
Israel	1	2.6%
Portugal	1	2.6%
Norway	1	2.6%
Sample size of analyses		
50–200	2	5.3%
201–500	4	10.5%
501–1000	5	13.1%
1001–5000	17	44.7%
5001–10,000	5	13.2%
Over 10,000	5	13.2%
Outcomes		
Inflammation	11	28.9%
Glucose metabolism	11	28.9%
Cardiovascular function	6	15.8%
Adiposity	5	13.1%
Lipid metabolism	5	13.1%
Allostatic load	4	10.5%
Parental socio‐economic position		
Parental education	12	31.5%
Parental occupation (both parents)	9	23.6%
Parental SEP (Parental education and occupation)	7	18.4%
Father's occupation	5	13.1%
Parental SEP	3	7.9%
Mother education	2	5.2%
Sex		
Men	3	7.9%
Women and men	35	92.1%
Age		
18–30	9	23.7%
31–60	16	42.1%
60 and above	9	23.7%
18 and older	4	10.5%
Ethnicity		
White participants	20	55.9%
Black participants	2	2.9%
White and nonwhite participants	16	41.2%

Parental SEP was operationalised through education (*n* = 19 studies, predominantly mother's education) and occupation (*n* = 21 studies). Outcome reporting emphasised inflammatory and glucose markers (*n* = 11), cardiovascular measures (*n* = 6), and lipid markers (*n* = 5), with the strongest evidence for blood pressure, adiposity and CRP outcomes. Most (81%) parental SEP data derived from survey instruments rather than registers.

Of the 38 studies assessed, 21 covering 69,774 participants had 60 estimates useful for meta‐analyses.^13^, ^14^, ^16^, ^19^, ^22^, ^38^, ^39^, ^40^, ^41^, ^42^, ^43^, ^44^, ^45^, ^46^, ^47^, ^48^, ^49^, ^50^, ^51^, ^52^, ^53^, ^54^

In nonpooled studies,^55^, ^56^, ^57^, ^58^, ^59^ lower SEP was consistently associated with adverse adult cardiometabolic outcomes, including elevated inflammatory outcomes, blood pressure, waist circumference, triglycerides, BMI and higher cholesterol risk. The reviewed studies comprised cohorts and cross‐sectional studies focusing on paediatric and adolescent populations (<18 years) and younger adults. Key limitations included inconsistent biomarker reporting and heterogeneous measurements of SEP and cardiometabolic outcomes.

### 3.2 Parental SEP and adult cardiometabolic and inflammatory outcomes

We found significant associations between lower parental SEP and offspring's cardiovascular function, adiposity, IL‐6, CRP and allostatic load levels in adulthood (Table 2).

**TABLE 2:** Summary of the random effects models reporting the intergenerational associations between low parental socio‐economic position and offspring cardiometabolic and inflammatory outcomes.

Single category	Outcomes	SMD^c^	CI	*I* ^2^ ^d^	CI
Cardiovascular function	Blood pressure (SBP and DBP)^a^	.30 mmHg	.10 to .50**	94.5%	92.0%–96.2%
Cardiovascular function	Blood pressure (SBP and DBP)^b^	2.76 mmHg	.10 to .50**	99.9%	99.8%–99.9%
Glucose metabolism	HbA1c, fasting glucose, glucose^a^	.77 units	−.22 to 1.76	99.8%	99.8%–99.9%
Lipid metabolism	Total cholesterol, triglyceride, LDL^a^	.05 mmol/L	−.40 to .31	99.6%	99.5%–99.6%
Adiposity	BMI and waist circumference^a^	.56 units	.05 to 1.07**	97.9%	97.2%–98.4%
Inflammation	C‐Reactive protein (CRP)^a^	1.45 mg/dl	.06 to 2.85**	99.6%	99.6%–99.7%
Inflammation	Interleukin‐6 (IL‐6)^a^	2.12 pg/mL	.72 to 4.97**	99.6%	99.5%–99.7%
Inflammation	CRP and IL‐6^b^	.33 units	.20 to .45***	75.2%	38.9%–89.9%
Multisystem dysregulation	Allostatic load^a^	.85 units	.30 to 1.40**	99.3%	99.1%–99.5%

*Note*: Significance levels ***p < .001, **p < .010, *p < .05.

^a^ Cohort studies.

^b^ Cross sectional studies.

^c^ SMD: standardised mean difference.

^d^ *I* ^2^: Quantifying heterogeneity.

### 3.3 Blood pressure

**FIGURE 2:**
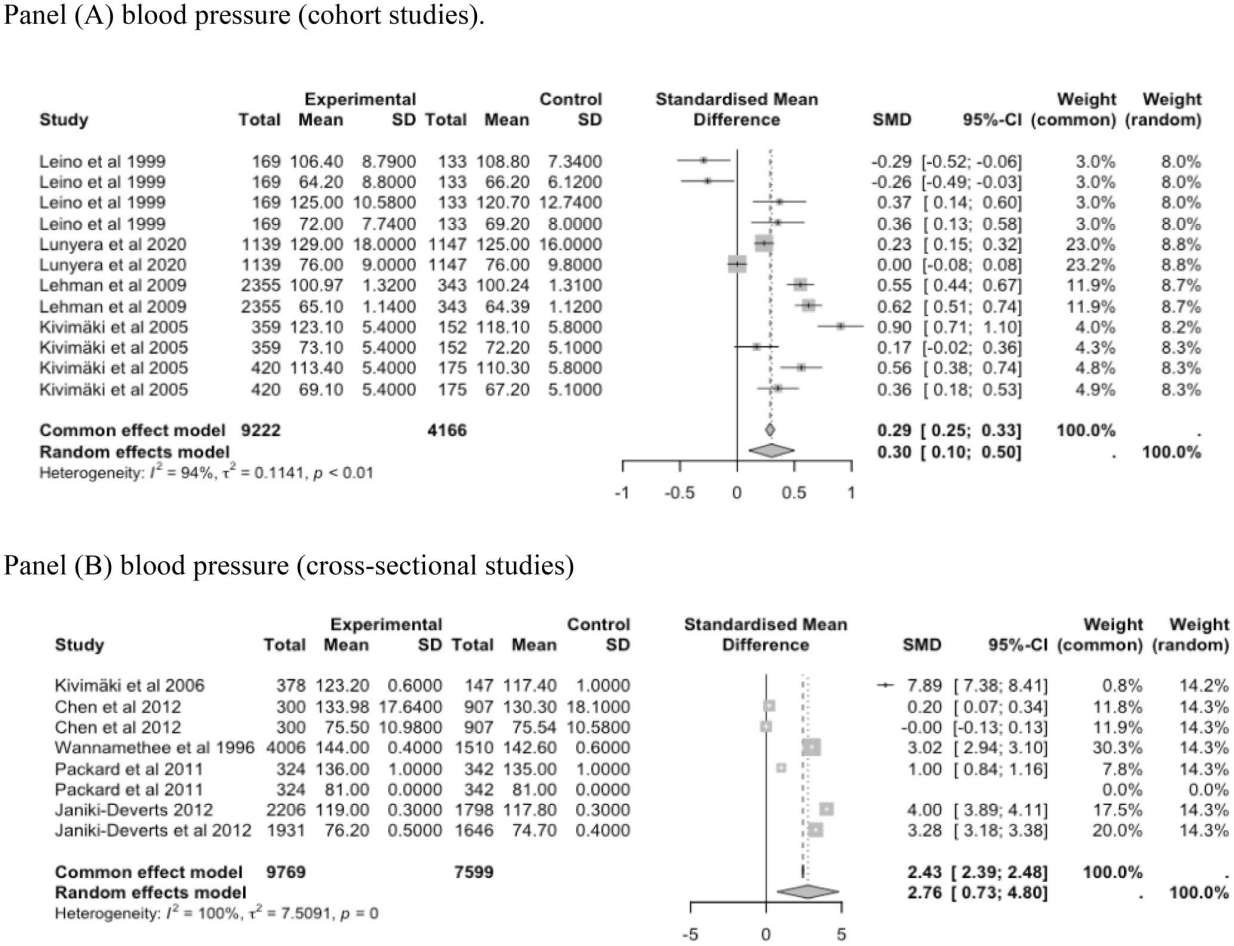
Random effects of the associations between low parental socio‐economic position and offspring's blood pressure in adulthood (cohort and cross‐sectional studies). (A) Blood pressure (cohort studies) and (B) blood pressure (cross‐sectional studies). Blood pressure: Estimates from Leino et al.^47^ and Kivimaki were reported separately for females and males for both SBP and DBP. Estimates from Lunyera et al.,^48^ Lehman et al.,^46^ Chen et al.,^60^ Packard et al.^16^ and Janicki‐Deverts et al.^43^ included combined data for both sexes for SBP and DBP.

The analysis indicated a significant association between lower parental socio‐economic position and higher blood pressure in later life in both cohort studies (Panel A, SMD = .30 mmHg; 95% CI, .10–.50, *I* ^2^=94%) and in cross‐sectional studies (Panel B, SMD = 2.76 mmHg; 95% CI, .10–.50, *I* ^2^=86%) (Figure 2).

### 3.4 Inflammatory outcomes and allostatic load

**FIGURE 3:**
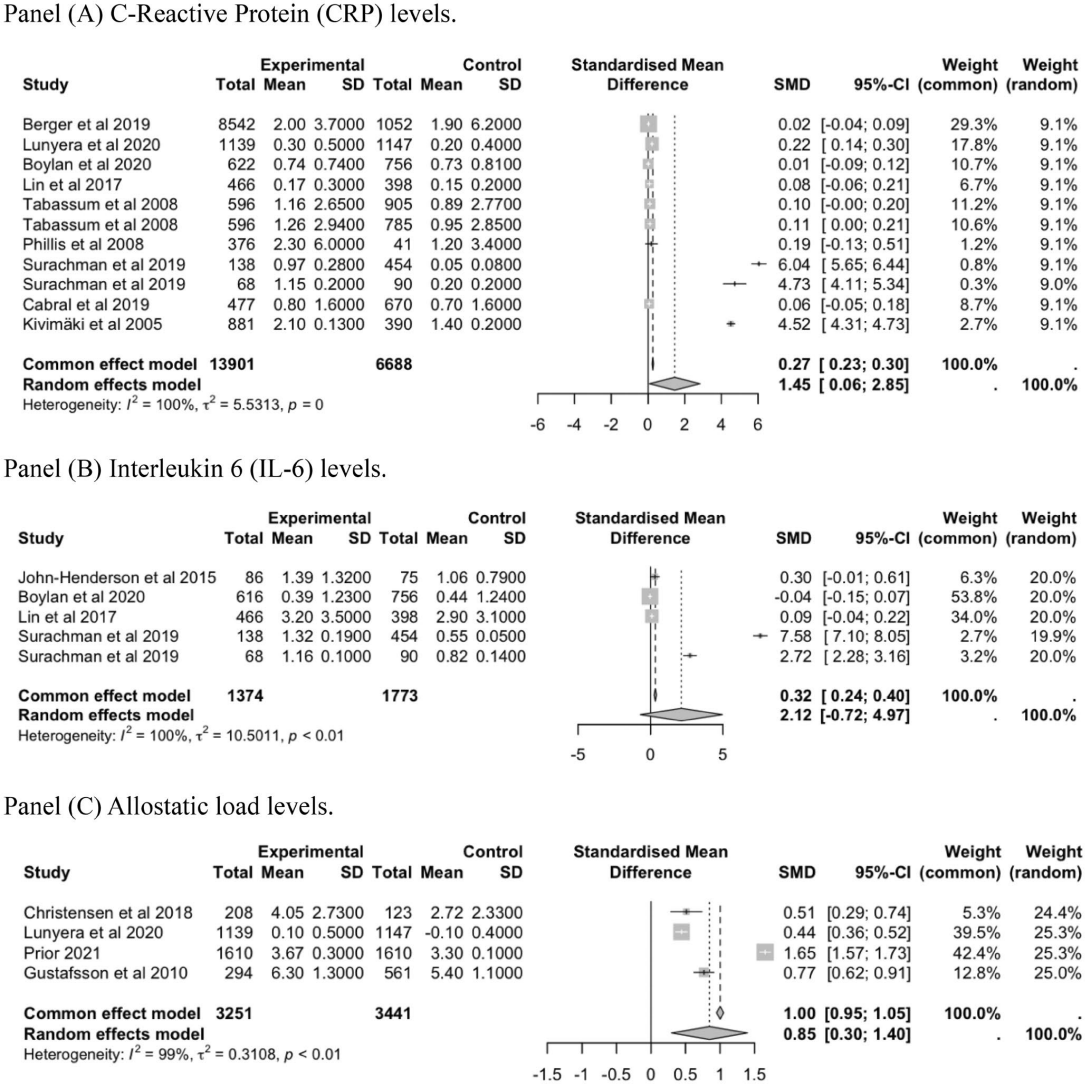
Random effects of the associations between low parental socio‐economic position (SEP) and offspring's C‐Reactive Protein (CRP), interleukin‐6 (IL‐6) and allostatic load levels in adulthood (cohort studies). (A) C‐reactive protein (CRP) levels, (B) interleukin 6 (IL‐6) levels and (C) allostatic load levels. C‐reactive protein and interleukin 6. Estimates from Tabassum et al.^53^ were reported separately for females and males for CRP. Estimates from Surachman et al.^73^ included combined data for both sexes for CRP and IL‐6.

Results from longitudinal data are presented in Figure 3. There was a consistent association between lower parental SEP and an increased risk of having higher levels of CRP (Panel A, SMD = 1.45 mg/dL; 95% CI, .06–2.85, I2=80%), IL‐6 (Panel B, SMD = 2.12 pg/mL; 95% CI, −.72 to 4.97, *I* ^2^=100%) and Allostatic load (Panel C, SMD = .85; 95% CI, .30–1.40, *I* ^2^=99%). In most of the studies, the AL score was computed using the traditional count‐based method of summing the number of allostatic load markers falling in the high‐risk quartile.^61^ Christensen et al.^13^, ^14^ used 14 markers representing the inflammatory, metabolic and cardiovascular systems measured at midlife. Lunyera et al.^48^ used 11 markers representing neuroendocrine, inflammatory, metabolic and cardiovascular systems. In the cross‐sectional studies, low parental SEP showed a significant association with inflammatory outcomes (SMD = .33; 95% CI, .20–.45, *I* ^2^=75%).

### 3.5 Metabolic outcomes

**FIGURE 4:**
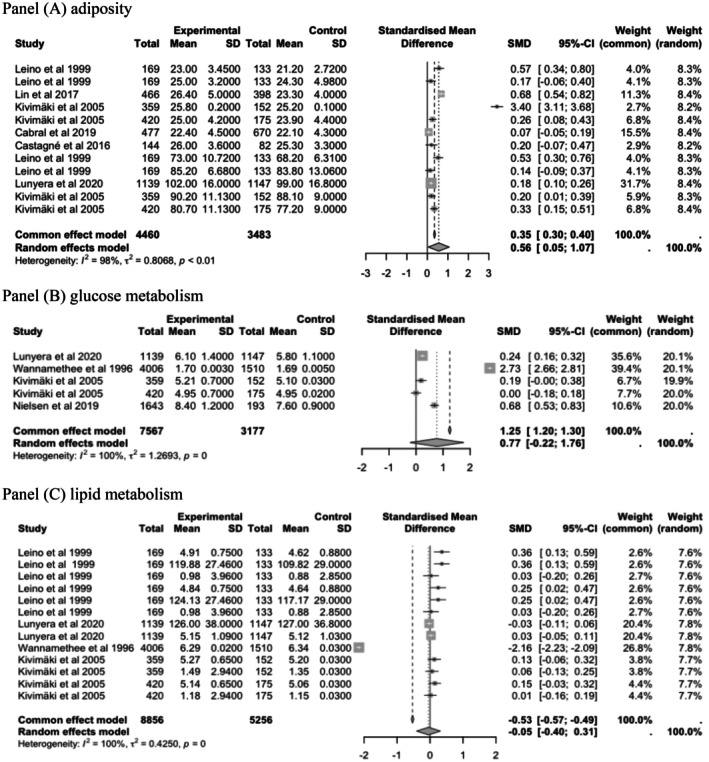
Random effects of the association between low parental socio‐economic position (SEP) and offspring's glucose metabolism in adulthood (cohort studies). (A) Adiposity: Estimates from Leino et al.^47^ and Kivimäki et al.^45^ were reported separately for females and males for both BMI and WC. (B) Glucose metabolism: Estimates from Kivimäki et al.^45^ were reported separately for males and females for fasting glucose for females and males. (C) Lipid metabolism: Estimates from Leino et al.^47^ were reported separately for females and males for total cholesterol, LDL cholesterol and triglycerides; Kivimäki et al.^45^ were reported separately for females and males for total cholesterol and triglycerides; estimates from Lunyera et al.^48^ included combined data for both sexes for SBP and DBP.

The analysis also indicated a significant association between lower parental SEP and adiposity (Figure 4 Panel A, SMD = .56; 95% CI, .05–1.07, *I* ^2^=98%), but not with glucose and lipid metabolism outcomes in cohort studies. Pooled estimates in panel A indicate that exposures to low parental SEP are associated with higher glucose metabolism (Panel A, SMD = .77 *I* ^2^=98%; 95% CI, −.22 to 1.76, *I* ^2^=100%) and higher adiposity levels (Panel B, SMD = .56; 95% CI, .05–1.07, *I* ^2^=98%). Parental SEP had a negligible effect on lipid metabolic biomarkers (Panel C, SMD = −.05 mmol/L; 95% CI, −.40 to .31, *I* ^2^=0%).

Subgroup analysis revealed substantial heterogeneity (*I* ^2^ > 79%) in associations between low parental SEP and elevated blood pressure, adiposity and C‐reactive protein (Table 3). Higher parental SEP was linked to better lipid metabolism in young adults (ages 21–30: SMD = .21, 95% CI .12–.30, *I* ^2^ = 39.5%); there was a marked CRP‐parental SEP association (SMD = 1.45, 95% CI .06–2.8, *I* ^2^ = 99.6%); and age‐stratified analysis showed significant blood pressure differences in the 24–39 years group (SMD = .50, 95% CI .21–.79). Meta‐regression confirmed subgroup variations (*p* < .0001). Lunyera^48^ disproportionately influenced pooled estimates. Funnel plot asymmetry suggested publication bias, although this should be interpreted with caution due to the limited number of studies and heterogeneity sources (age and gender differences). (Figure S1 funnel plots asymmetry).

**TABLE 3:** Subgroup analyses of association between parental SEP and adult cardiometabolic and inflammatory outcomes.

Subgroup analyses	*N*	Pooled SMD (95% CI)		*I* ^*2*^ (%)	*p* for heterogeneity	*p* for meta‐regression^a^
Blood pressure (cohort studies)						<.0001
Age 21–30 years	4	.04 [−.25; .34]		90.0	–	
Age 24–39 years	4	.49 [.20; .78]		90.2	–	
Blood pressure (cross‐sectional)	7	2.76 [.73; 4.79]		99.9	0	<.0001
Glucose metabolism (cohort studies)	5	.76 [−.21; 1.75]		99.8	0	<.0001
Lipid metabolism (cohort studies)						0
Age 21–30 years	6	.20 [.11; .30]		39.5	<.0001	
Age 24–39 years	4	.08 [−.00; .17]		.0	0	
Adiposity (cohort studies)	4	.28 [−.56; 1.13]		93.9	<.0001	.2880
CRP (cohort studies)	11	1.45 [.06; 2.84]		99.6	0	<.0001
IL‐6 (cohort studies)	5	2.12 [−.72; 4.96]		99.6	<.0001	.4602
Inflammation (cohort studies)	4	.84 [.29; 1.39]		99.3	<.0001	<.0001
Allostatic load (cross sectional)	5	.32 [.20; .45]		75.2	.0029	.0193

*Note*: Blood pressure (SBP and DBP). Glucose metabolism (HbA1c, fasting glucose, blood glucose). Lipid metabolism based on cohort studies (total cholesterol, triglyceride and LDL). This study did not include HDL. Inflammation (CRP and IL‐6). Adiposity (BMI and WC). Interpretation of heterogeneity levels: *I* ^2^ = 25%: low heterogeneity; *I* ^2^ = 50%: moderate heterogeneity and *I* ^2^ = 75%: substantial heterogeneity.

^a^ Meta‐regression *p*‐values test subgroups differences.

## 4 DISCUSSION

Our results suggest that children exposed to lower parental socio‐economic position have a higher risk of elevated levels of CRP, IL‐6, allostatic load and an increased risk of adiposity in later adulthood compared to children with higher parental socio‐economic position. This aligns with findings from Slopen et al.,^6^ Liu et al.^5^ and Milaniak and Jaffee,^62^ which linked exposure to lower parental socio‐economic position to higher levels of C‐reactive protein and interleukin‐6. Liu et al.'s^5^ meta‐analysis using 15 observational studies found that, compared with those from the most advantaged families, individuals from the least advantaged families had 25% higher odds of elevated CRP in later life (OR = 1.25; 95% CI 1.19, 1.32). Inflammatory findings seem to be strongly driven by a single study.^48^ This study was different from the others in the exposure variable used, cumulative life‐course socio‐economic position. In this study, participants' self‐reported childhood socio‐economic status, educational attainment and annual household income. This methodological distinction suggests that cumulative SEP may better capture biological embedding than single‐point assessments or that self‐reported childhood SEP could inflate effect sizes.

The finding of a significant association between lower parental socio‐economic position and risk of adiposity in later adulthood is inconsistent with the Senese et al.^63^ systematic review, while Newton et al.^64^ found a link between lower SEP and higher obesity in females, based mostly on studies in developed countries. Tamayo et al.,^7^ Parsons et al.^65^ and Gonzalez et al.^66^ systematic reviews, and Slopen et al.^6^ meta‐analysis also reported an association between low SEP in early life and greater central adiposity in adulthood.

Mechanisms of association between parental SEP and adult adiposity may be through the development of adiposity in childhood/adolescence, which then persists through adult life. Or it may be through the mediating role of adult SEP, influenced by parental and childhood SEP. The observed inverse relationship between parental SEP and adiposity, mainly among women, was based primarily on studies in high‐income countries.^64^, ^66^, ^67^

In our study, parental SEP was associated with higher allostatic load in adulthood, ranging from 10% to 14%. This result aligns with findings from Finlay et al.^68^ and Misiak et al.,^69^ which systematically reviewed 18 studies mainly focused on the US and European samples. They found evidence of an association with elevated allostatic load indices in adulthood. However, markers and biological systems included in the AL index differed widely across studies. Despite these differences, pooled studies showed significant associations between lower parental socio‐economic position and elevated allostatic load.

Lower parental socio‐economic position was linked to a modest risk of elevated blood pressure. Cross‐sectional studies indicated larger effects of parental SEP on adult blood pressure compared to cohort studies. A potential reason for the difference in the magnitude of the effects between the two study designs may be related to confounding variables, selection bias and methodological differences (assessment of SEP measures or assessment of blood pressure) that could be influencing this difference.

Our findings are consistent with McHutchison et al.^70^ meta‐analysis using 10 cohort studies from the US, Denmark, UK, Sweden and Scotland, which found modest associations between parental SEP and lifetime cardiovascular risks. In the Mallinson et al.^71^ meta‐analysis, 38 out of the 46 studies showed no clear evidence of association with the risk of elevated blood pressure. This meta‐analysis found more studies than ours as it was mainly based on investigations from middle‐income countries with differences in methodology, scope and search strategy, which significantly influenced the number of the studies included. In addition, inconsistencies in the findings may be related to the differences in blood pressure measurements, parental SEP measures and study populations.

More imprecise estimates and mostly null effects were found for glucose metabolism (HbA1c, fasting glucose and glucose) and lipid metabolism markers (total cholesterol, triglyceride and LDL), suggesting no association with parental SEP. One primary explanation is due to the lack of statistical power in our meta‐analysis. These weaker associations have been previously documented by others^6^, ^72^: both systematic reviews were inconclusive on the association between childhood stressors and lipids or carbohydrate metabolism‐related factors.

In summary, several factors can explain the variation in effect sizes for markers, including differences in the study design, sample sizes, methods, markers and exposure collection procedures, and reported metrics (unadjusted and adjusted odds ratios, regression coefficients, relative risk ratios, path coefficients from structural equation models, mean differences).

The strongest association in the subgroup analysis highlights that parental SEP and lipid metabolism and parental SEP and CRP might show stronger associations in young adults. Moreover, age matters in these associations as effects vary by life stage. Most outcomes show high between‐study variability (*I* ^2^ > 90%). This high heterogeneity indicates substantial variability in the effect sizes across included studies. This considerable heterogeneity has significant implications for interpreting the results. It suggests that the generalisability of these findings is limited as the observed intergenerational associations may vary considerably depending on specific study populations, methodologies and contexts. Therefore, caution is warranted when applying these aggregated results to different populations, and further research is needed to understand the sources of this heterogeneity and to identify subgroups where these intergenerational associations are more consistent.

Possible sources of asymmetry in funnel plots may include reporting biases and publication bias, poor methodological elements leading to spuriously inflated effects in smaller studies, true heterogeneity (effect sizes differ according to study size) or changes in reported outcomes.^35^ Therefore, the limited number of studies in this meta‐analysis impedes a conclusive assessment.

This study was subject to several limitations. Most included studies did not provide disaggregated data by characteristics such as age, gender, race and ethnic groups. Therefore, our systematic review did not investigate the extent to which the associations differed for men versus women, for white versus black populations, for Latino versus European populations, etc. Secondly, collapsing parental SEP into a single measure did not allow the investigation of how different parental SEP measures such as mother's education or father's occupation differed across markers.

Thirdly, our meta‐analysis was not large enough to make a definitive claim about the associations investigated. In addition, the evidence mainly comes from HIC and the findings are not generalisable to low‐ and middle‐income populations.

Fourth, the study of associations between parental SEP and glucose and lipid metabolic markers needs more statistical power. Although collapsing physiological related outcomes improved power, sometimes grouping some outcomes could obscure effects. Despite our search was extensive through different search engines and carried out by three trained independent reviewers, we might have missed relevant studies addressing our research question. Finally, it is possible that there are further confounders and mediators behind the observed relationships. The biological pathways are not well established yet in the literature, and this can influence future research directions.

## 5 CONCLUSIONS

Our meta‐analysis suggests that low parental socio‐economic position negatively impacts adult offspring health, manifesting as higher blood pressure, elevated C‐reactive protein, increased interleukin‐6, greater adiposity and higher allostatic load. These findings align with evidence on the long‐term effects of early life circumstances on health outcomes. Future research should prioritise three critical areas: (1) Mechanistic specificity (deconstructing SEP measures to understand how parental education and occupation affect biological mechanisms); (2) Intersectional pathways (investigating pathways and differences across gender, race and region); and (3) life‐course timing and critical period detection. Researchers could aim to determine whether adolescence is a sensitive period for metabolic dysregulation and to assess how lower parental SEP also tends to lead to higher exposure to adverse childhood experiences (ACEs). The timing of health outcome development, differentiating direct from indirect effects mediated by adult individual SEP, is also a goal for future research. Identifying these causal pathways should be at the basis of the next generation research.

## AUTHOR CONTRIBUTIONS

JCR, EC, and PV were involved in conceptualization. JCR, OAC, RD, and OMM were involved in data curation. JCR was involved in formal analysis, project administration, visualization, and writing—original draft. JCR and PV were involved in funding acquisition. JCR, EC, PV, and EW were involved in investigation. JCR, EC, EW, and PV were involved in methodology and Writing—review and editing. EC, EW, and PV were involved in supervision. JCR, OAC, EC, and EW were involved in validation.

## CONFLICT OF INTEREST STATEMENT

All authors declare that they have no conflicts of interest.

## Supporting information


Data S1.

## Data Availability

Relevant meta‐level data, protocol and analytical code on which this analysis is based are available on request to the corresponding author (AEP).

## References

[1] Turner RJ , Thomas CS , Brown TH . Childhood adversity and adult health: evaluating intervening mechanisms. Soc Sci Med. 2016;156:114‐124. doi:10.1016/j.socscimed.2016.02.026 27030896

[2] Vineis P , Avendano‐Pabon M , Barros H , et al. Special report: The biology of inequalities in health: The lifepath consortium. Front Public Health. 2020;8:1‐37. doi:10.3389/fpubh.2020.00118 32478023 PMC7235337

[3] Walsh D , McCartney G , Smith M , Armour G . Relationship between childhood socioeconomic position and adverse childhood experiences (ACEs): a systematic review. J Epidemiol Community Health. 2019;73(12):1087‐1093. doi:10.1136/jech-2019-212738 31563897 PMC6872440

[4] King AL . Understanding Relationships between Early Life Toxic Stress, Childhood Socioeconomic Disadvantage, and Allostatic Load in Adolescence. In *ProQuest Dissertations and Theses* (Issue August). 2018. https://search.proquest.com/dissertations‐theses/understanding‐relationships‐between‐early‐life/docview/2094606775/se‐2?accountid=41304

[5] Liu RS , Aiello AE , Mensah FK , et al. Socioeconomic status in childhood and C reactive protein in adulthood: a systematic review and meta‐analysis. J Epidemiol Community Health. 2017;71(8):817‐826. doi:10.1136/jech-2016-208646 28490476 PMC5843476

[6] Slopen N , Goodman E , Koenen KC , Kubzansky LD . Socioeconomic and other social stressors and biomarkers of cardiometabolic risk in youth: a systematic review of less studied risk factors. PLoS One. 2013;8:e64418. doi:10.1371/journal.pone.0064418 23691213 PMC3656855

[7] Tamayo T , Herder C , Rathmann W . Impact of early psychosocial factors (childhood socioeconomic factors and adversities) on future risk of type 2 diabetes, metabolic disturbances and obesity: a systematic review. Holistic Perspectives on Trauma. Apple Academic Press; 2015:315‐340. doi:10.1201/b18313-20 PMC2940917 20809937

[8] Von Kobyletzki LB , Beckman L , Smeeth L , et al. Association between childhood allergic diseases, educational attainment and occupational status in later life: systematic review protocol. BMJ Open. 2017;7(10):1‐4. doi:10.1136/bmjopen-2017-017245 PMC5652581 29025838

[9] Borenstein AR , Copenhaver CI , Mortimer JA . Early‐life risk factors for Alzheimer disease. Alzheimer Dis Assoc Disord. 2006;20(1):63‐72. doi:10.1097/01.wad.0000201854.62116.d7 16493239

[10] Calvin CM , Batty GD , Lowe GDO , Deary IJ . Childhood intelligence and midlife inflammatory and hemostatic biomarkers: the National Child Development Study (1958) cohort. Health Psychol. 2011;30(6):710‐718. doi:10.1037/a0023940 21604878

[11] Klemfuss JZ , Olaguez AP . Individual differences in Children's suggestibility: an updated review. J Child Sex Abus. 2020;29(2):158‐182. doi:10.1080/10538712.2018.1508108 30142291

[12] Case A , Fertig A , Paxson C . The lasting impact of childhood health and circumstance. J Health Econ. 2005;24(2):365‐389. doi:10.1016/j.jhealeco.2004.09.008 15721050

[13] Christensen DS , Flensborg‐Madsen T , Garde E , Hansen ÅM , Pedersen JM , Mortensen EL . Parental socioeconomic position and midlife allostatic load: a study of potential mediators. BMC Public Health. 2018;18(1):1‐11. doi:10.1186/s12889-018-5956-x PMC6102839 30126406

[14] Christensen DS , Flensborg‐Madsen T , Garde E , Hansen ÅM , Pedersen JM , Mortensen EL . Early life predictors of midlife allostatic load: a prospective cohort study. PLoS One. 2018;13(8):1‐15. doi:10.1371/journal.pone.0202395 PMC6095582 30114237

[15] Bennett NR , Ferguson TS , Bennett FI , et al. High‐sensitivity C‐reactive protein is related to central obesity and the number of metabolic syndrome components in Jamaican Young adults. Front Cardiovasc Med. 2014;1(December):1‐9. doi:10.3389/fcvm.2014.00012 PMC4668855 26664862

[16] Packard CJ , Bezlyak V , McLean JS , et al. Early life socioeconomic adversity is associated in adult life with chronic inflammation, carotid atherosclerosis, poorer lung function and decreased cognitive performance: a cross‐sectional, population‐based study. BMC Public Health. 2011;11:42. doi:10.1186/1471-2458-11-42 21241479 PMC3032683

[17] Campbell TS , Key BL , Ireland AD , Bacon SL , Ditto B . Early socioeconomic status is associated with adult nighttime blood pressure dipping. Psychosom Med. 2008;70(3):276‐281. doi:10.1097/PSY.0b013e3181647e30 18256336

[18] Kivimäki M , Davey Smith G , Juonala M , et al. Socioeconomic position in childhood and adult cardiovascular risk factors, vascular structure, and function: cardiovascular risk in young Finns study. Heart. 2006;92(4):474‐480. doi:10.1136/hrt.2005.067108 16159979 PMC1860895

[19] Kivimäki M , Smith GD , Elovainio M , et al. Socioeconomic circumstances in childhood and blood pressure in adulthood: the cardiovascular risk in Young Finns study. Ann Epidemiol. 2006;16(10):737‐742. doi:10.1016/j.annepidem.2006.01.004 16843680

[20] Cohen S , Janicki‐Deverts D , Chen E , Matthews KA . Childhood socioeconomic status and adult health. Ann N Y Acad Sci. 2010;1186(1):37‐55. doi:10.1111/j.1749-6632.2009.05334.x 20201867

[21] Franceschi C , Campisi J . Chronic inflammation (inflammaging) and its potential contribution to age‐associated diseases. J Gerontol A Biol Sci Med Sci. 2014;69:S4‐S9. doi:10.1093/gerona/glu057 24833586

[22] Lin YH , Jen MH , Chien KL . Association between life‐course socioeconomic position and inflammatory biomarkers in older age: a nationally representative cohort study in Taiwan. BMC Geriatr. 2017;17(1):1‐11. doi:10.1186/s12877-017-0598-x 28865434 PMC5581430

[23] Miller GE , Chen E , Parker KJ . Psychological stress in childhood and susceptibility to the chronic diseases of aging: moving toward a model of behavioral and biological mechanisms. Psychol Bull. 2011;137(6):959‐997. doi:10.1037/a0024768 21787044 PMC3202072

[24] Shanahan L , Freeman J , Bauldry S . Is very high C‐reactive protein in young adults associated with indicators of chronic disease risk? Psychoneuroendocrinology. 2014;40(1):76‐85. doi:10.1016/j.psyneuen.2013.10.019 24485478 PMC4307946

[25] Colich NL , Rosen ML , Williams ES , Mclaughlin KA . Biological aging in childhood and adolescence following experiences of threat and deprivation: a systematic review and meta‐analysis HHS public access. Psychol Bull. 2020;146(9):721‐764. doi:10.1037/bul0000270 32744840 PMC7484378

[26] Soares S , Rocha V , Kelly‐Irving M , Stringhini S , Fraga S . Adverse childhood events and health biomarkers: a systematic review. Front Public Health. 2021;9:649825. doi:10.3389/fpubh.2021.649825 34490175 PMC8417002

[27] Borenstein M , Hedges LV , Higgins JPT , Rothstein HR . Introduction to Meta‐Analysis. John Wiley & Sons; 2009:1‐421. doi:10.1002/9780470743386

[28] Higgins JPT , Thomas J , Chandler J , et al. Cochrane Handbook for Systematic Reviews of Interventions. Cochrane; 2019:258‐273.

[29] Farrah K , Young K , Tunis MC , Zhao L . Risk of bias tools in systematic reviews of health interventions: an analysis of PROSPERO‐registered protocols. Syst Rev. 2019;8(1):1‐9. doi:10.1186/s13643-019-1172-8 31730014 PMC6857304

[30] World Health Organization (WHO) . Diagnosis and classification of diabetes mellitus. Diabetes Care. 2010;33(Supplement_1):S62‐S69. doi:10.2337/dc10-S062 20042775 PMC2797383

[31] National Heart, Lung, Blood Institute, National Institute of Diabetes, Digestive, Kidney Diseases (US) . Clinical Guidelines on the Identification, Evaluation, and Treatment of Overweight and Obesity in Adults. National Heart, Lung, and Blood Institute; 1998. https://www.ncbi.nlm.nih.gov/books/NBK2003/

[32] Cheung MWL , Vijayakumar R . A guide to conducting a meta‐analysis. Neuropsychol Rev. 2016;26(2):121‐128. doi:10.1007/s11065-016-9319-z 27209412

[33] Borenstein M , Wiley J . How a Meta‐Analysis Works. Wiley; 2009.

[34] Hak T , van Rhee H , Suurmond R . How to interpret results of meta‐ analysis. 2018.

[35] Sterne JAC , Sutton AJ , Ioannidis JPA , et al. Recommendations for examining and interpreting funnel plot asymmetry in meta‐analyses of randomised controlled trials. BMJ. 2011;343(7818):d4002. doi:10.1136/BMJ.D4002 21784880

[36] Pipis G . Meta Analysis In R. Example of Meta‐Analysis using R. 2023 https://medium.com/geekculture/meta‐analysis‐in‐r‐f75715f30162

[37] Quintana D . Calculating the statistical power of studies included in a meta‐analysis using the {metameta} R package (No. 12761885). 17. none. 2021.

[38] Berger E , Castagné R , Chadeau‐Hyam M , et al. Multi‐cohort study identifies social determinants of systemic inflammation over the life course. Nat Commun. 2019;10(1):773. doi:10.1038/s41467-019-08732-x 30770820 PMC6377676

[39] Boylan JM , Cundiff JM , Fuller‐Rowell TE , Ryff CD . Childhood socioeconomic status and inflammation: psychological moderators among Black and White Americans. Health Psychol. 2020;39(6):497‐508. doi:10.1037/hea0000866 32212770 PMC7437114

[40] Cabral M , Severo M , Barros H , Guimarães JT , Ramos E . Longitudinal association of adiposity and high‐sensitivity C‐reactive protein from adolescence into early adulthood. Nutr Metab Cardiovasc Dis. 2019;29(6):590‐597. doi:10.1016/j.numecd.2019.03.008 31078361

[41] Castagné R , Kelly‐irving M , Campanella G , et al. Biological marks of early‐life socioeconomic experience is detected in the adult inflammatory transcriptome. Nature. 2016;6:1‐10. 10.1038/srep38705 PMC5146729 27934951

[42] Gustafsson PE , Janlert U , Theorell T , Westerlund H , Hammarström A . Socioeconomic status over the life course and allostatic load in adulthood: results from the northern Swedish cohort. J Epidemiol Community Health. 2011;65(11):986‐992. doi:10.1136/jech.2010.108332 20974835

[43] Janicki‐Deverts D , Cohen S , Matthews KA , Jacobs DR . Sex differences in the association of childhood socioeconomic status with adult blood pressure change: the CARDIA study. Psychosom Med. 2012;74(7):728‐735. doi:10.1097/PSY.0b013e31825e32e8 22822232 PMC3434230

[44] John‐Henderson NA , Marsland AL , Kamarck TW , Muldoon MF , Manuck SB . Childhood socioeconomic status and the occurrence of recent negative life events as predictors of circulating and stimulated levels of interleukin‐6. Psychosom Med. 2016;78(1):91‐101. doi:10.1097/PSY.0000000000000262 26727383 PMC4700553

[45] Kivimäki M , Lawlor DA , Juonala M , et al. Lifecourse socioeconomic position, C‐reactive protein, and carotid intima‐media thickness in young adults: the cardiovascular risk in Young Finns Study. Arterioscler Thromb Vasc Biol. 2005;25(10):2197‐2202. doi:10.1161/01.ATV.0000183729.91449.6e 16123322

[46] Lehman BJ , Taylor SE , Kiefe CI , Seeman TE . Relationship of early life stress and psychological functioning to blood pressure in the CARDIA Study. Health Psychol. 2009;28(3):338‐346. doi:10.1037/a0013785 19450040 PMC2844101

[47] Leino M , Raitakari OT , Porkka KVK , Taimela S , Viikari JSA . Associations of education with cardiovascular risk factors in young adults: the cardiovascular risk in Young Finns Study. Int J Epidemiol. 1999;28(4):667‐675. doi:10.1093/ije/28.4.667 10480694

[48] Lunyera J , Stanifer JW , Davenport CA , et al. Life course socioeconomic status, allostatic load, and kidney health in black americans. Clin J Am Soc Nephrol. 2020;15(3):341‐348. doi:10.2215/CJN.08430719 32075808 PMC7057315

[49] Nielsen NF , Gaulke A , Eriksen TM , Svensson J , Skipper N . Socioeconomic inequality in metabolic control among children with type 1 diabetes: a Nationwide longitudinal study of 4,079 Danish children. Diabetes Care. 2019;42(8):1398‐1405. doi:10.2337/dc19-0184 31123155

[50] Präg P , Richards L . Intergenerational social mobility and allostatic load in Great Britain. J Epidemiol Community Health. 2019;73(2):100‐105. doi:10.1136/jech-2017-210171 30385515

[51] Prior L . Allostatic load and exposure histories of disadvantage. Int J Environ Res Public Health. 2021;18:7222. doi:10.3390/ijerph18147222 34299672 PMC8308019

[52] Surachman A , Rice C , Bray B , Gruenewald T , Almeida D . Association between socioeconomic status mobility and inflammation markers among white and black adults in the United States: a latent class analysis. Psychosom Med. 2020;82(2):224‐233. doi:10.1097/PSY.0000000000000752 31592888 PMC7007866

[53] Tabassum F , Kumari M , Rumley A , Lowe G , Power C , David P . Original contribution effects of socioeconomic position on inflammatory and hemostatic markers: a life‐course analysis in the 1958 British birth cohort. 2008;167(11):1332‐1341. doi:10.1093/aje/kwn055 18367468

[54] Wannamethee SG , Whincup PH , Shaper G , Walker M . Influence of fathers' social class on cardiovascular disease in middle‐aged men. Lancet. 1996;348(9037):1259‐1263. doi:10.1016/S0140-6736(96)02465-8 8909377

[55] Brunner E , Smith GD , Marmot M , Canner R , Beksinska M , O'Brien J . Childhood social circumstances and psychosocial and behavioural factors as determinants of plasma fibrinogen. Lancet. 1996;347(9007):1008‐1013. doi:10.1016/S0140-6736(96)90147-6 8606563

[56] Carroll JE , Gruenewald TL , Taylor SE , Janicki‐Deverts D , Matthews KA , Seeman TE . Childhood abuse, parental warmth, and adult multisystem biological risk in the coronary artery risk development in young adults study. Proc Natl Acad Sci USA. 2013;110(42):17149‐17153. doi:10.1073/pnas.1315458110 24062432 PMC3800991

[57] Gustafsson PE , Janlert U , Theorell T , Hammarström A . Life‐course socioeconomic trajectories and diurnal cortisol regulation in adulthood. Psychoneuroendocrinology. 2010;35(4):613‐623. doi:10.1016/j.psyneuen.2009.09.019 19879057

[58] Heshmati A , Mishra G , Koupil I . Childhood and adulthood socio‐economic position and hypertensive disorders in pregnancy: the uppsala birth cohort multigenerational study. J Epidemiol Community Health. 2013;67(11):939‐946. doi:10.1136/jech-2012-202149 23729327

[59] Kvaavik E , Glymour M , Klepp KI , Tell GS , Batty GD . Parental education as a predictor of offspring behavioural and physiological cardiovascular disease risk factors. Eur J Pub Health. 2012;22(4):544‐550. doi:10.1093/eurpub/ckr106 21893507 PMC3402716

[60] Chen E , Miller GE , Lachman ME , Gruenewald TL , Seeman TE . Protective factors for adults from low childhood socioeconomic circumstances: the benefits of shift‐and‐persist for allostatic load. Psychosom Med. 2012;74(2):178‐186. doi:10.1097/PSY.0b013e31824206fd 22286848 PMC3273596

[61] Seeman T , Epel E , Gruenewald T , Karlamangla A , McEwen BS . Socio‐economic differentials in peripheral biology: cumulative allostatic load. Ann N Y Acad Sci. 2010;1186:223‐239. doi:10.1111/j.1749-6632.2009.05341.x 20201875

[62] Milaniak I , Jaffee SR . Childhood socioeconomic status and inflammation: a systematic review and meta‐analysis. Brain Behav Immun. 2019;78:161‐176. doi:10.1016/j.bbi.2019.01.018 30738842

[63] Senese LC , Almeida ND , Fath AK , Smith BT , Loucks EB . Associations between childhood socioeconomic position and adulthood obesity. Epidemiol Rev. 2009;31(1):21‐51. doi:10.1093/epirev/mxp006 19648176 PMC2873329

[64] Newton S , Braithwaite D , Akinyemiju TF . Socio‐economic status over the life course and obesity: systematic review and meta‐analysis. PLoS One. 2017;12(5):e0177151. doi:10.1371/journal.pone.0177151 28510579 PMC5433719

[65] Parsons TJ , Power C , Logan S , Summerbell CD . Childhood predictors of adult obesity: a systematic review. Int J Obes. 1999;23(SUPPL. 8):S1‐S107. 10641588

[66] González D , Nazmi A , Victora CG . Childhood poverty and abdominal obesity in adulthood: a systematic review. Cad Saude Publica. 2009;25(suppl 3):S427‐S440. doi:10.1590/s0102-311x2009001500008 20027390

[67] Chao C‐Y , Shih C‐C , Wang C‐J , et al. Low socioeconomic status may increase the risk of central obesity in incoming university students in Taiwan. Obes Res Clin Pract. 2014;8(3):e201‐e298. doi:10.1016/j.orcp.2012.07.002 24847662

[68] Finlay S , Roth C , Zimsen T , Bridson TL , Sarnyai Z , McDermott B . Adverse childhood experiences and allostatic load: a systematic review. Neurosci Biobehav Rev. 2022;136:104605. doi:10.1016/j.neubiorev.2022.104605 35278597

[69] Misiak B , Stańczykiewicz B , Pawlak A , et al. Adverse childhood experiences and low socioeconomic status with respect to allostatic load in adulthood: a systematic review. Psychoneuroendocrinology. 2022;136:105602. doi:10.1016/j.psyneuen.2021.105602 34861465

[70] McHutchison CA , Backhouse EV , Cvoro V , Shenkin SD , Wardlaw JM . Education, socioeconomic status, and intelligence in childhood and stroke risk in later life: a meta‐analysis. Epidemiology. 2017;28(4):608‐618. 28410350 10.1097/EDE.0000000000000675

[71] Mallinson PAC , Lieber J , Kinra S . Childhood socioeconomic position and risk of cardiovascular disease in adulthood: systematic review of evidence from low‐ and middle‐income countries. Am J Prev Med. 2021;61(5):e251‐e266. doi:10.1016/j.amepre.2021.04.027 34272136

[72] Jakubowski KP , Cundiff JM , Matthews KA . Supplemental material for cumulative childhood adversity and adult cardiometabolic disease: a meta‐analysis. Health Psychol. 2018;37(8):701‐715. doi:10.1037/hea0000637.supp 30024227 PMC6109976

[73] Surachman A, , Wardecker B, , Chow SM , Almeida D . Life Course Socioeconomic Status, Daily Stressors, and Daily Well‐Being: Examining Chain of Risk Models. J Gerontol B Psychol Sci Soc Sci. 2019 Jan 1;74(1):126‐135. doi:10.1093/geronb/gby014 29669043 PMC6294233

