# Supplementary material for: The intergenerational effects of low parental socio‐economic position on cardiometabolic and inflammatory outcomes: A systematic review and meta‐analysis
Source: Eur J Clin Invest. 2025 Sep 29;56(1):e70125. doi: 10.1111/eci.70125 (PMC12817245; doi:10.1111/eci.70125)

**Supplementary material**

**The Intergenerational Effects of Low Parental Socioeconomic Position on Cardiometabolic and Inflammatory Outcomes: A Systematic Review and Meta-analysis**

| **Appendix S1** | **Search strategy** |
| --- | --- |
| **Supplementary Table 1** | **The characteristics of the included individual studies** |
| **Supplementary Figure 1** | **Funnel plot asymmetry** |

**Appendix S1. Search strategy.**

Search included MESH terms and other relevant words and expressions using Boolean logic ("AND" or "OR") differently:

Search terms for **parental SES** includes:

Parental education

Parental schooling

Parental inequalities

Parental socioeconomic position

Parental investment

Parental occupation

Parental SES/SEP

Parental position

Parental disadvantage

Parental adversity

Parental circumstances

Parental conditions

Parental environment

Parental disparities

Parental experience

Parental factors

Parental determinants

Parental social class

Search terms for the **outcome** includes:

C-reactive protein

Blood pressure

Cardiovascular system

Cardiovascular biomarkers

Chronic kidney disease

Creatinine clearance

Cholesterol

Fibrinogen

Inflammation biomarkers

Inflammation

Inflammatory dysregulation

Inflammatory diseases

Inflammaging

Pro-inflammatory

Interleukin-6

Cardiometabolic disease

Cardiometabolic markers

Chronic cardiometabolic

Chronic inflammation

Metabolic system

Metabolic response

Metabolic dysregulation

Metabolic biomarkers

Metabolic diseases

Metabolism

Renal function

Kidney function

The reference list for the **population** includes:

Adult, adulthood, early adulthood, later life, life course, mid-adulthood, and older ages.

Ovid databases (Embase)

For question **are chronic cardiometabolic and inflammatory disease affected by the SES in later life?**the search strategy was:

1. All the keywords and subject headings for **adult biomarkers** combined with OR
2. All the keywords and subject headings for adult **inflammation** combined with AND
3. All the keywords and subject headings for **parental inequalities** combined with OR
4. Proximity searching using **parental* adj3 status***
5. 1 and 2 and 3 and 4

A detailed search strategy with combination of truncation and keywords is listed below with a specific example. Automatically does phrase searching e.g., **C-reactive protein** will only find those two words together in that order. The proximity Searching allows to search:

Adult* adj1 **C-reactive protein*** did find those two words together in either order (so could find **adult C-reactive protein** or **C-reactive protein adult**).

**Adult*** adj2 **C-reactive protein*** did find those two words together in either order or also those two words with another word in between (so could find **C-reactive protein in adult**).

**Adult*** adj3 **C-reactive protein*** did find those two words together in either order or also those two words with up to two other words in between etc.

The databases did not suggest subject headings if uses adj and may be confused by truncation for keywords.

Embase Classic+Embase <1947 to 2021 Week 21>

1 (parental education or parental schooling or parental inequalities or parental circumstances or parental conditions or parental investment or parental occupation or parental social environment or parental disparities or parental SES or parental position or parental disadvantage or Parental adversity or Parental experience).af. 6996

2 limit 1 to yr="1980 -Current" 6895

3 (((adult* adj3 biomarker*).af. or adult*.mp.) adj3 C-reactive protein*.af.) or CRP.af. or blood pressure.af. or cardiovascular biomarkers.af. or chronic kidney disease.af. or creatinine clearance.af. or cholesterol.af. or fibrinogen.af. or inflammation biomarker.af. or inflammation.af. or inflammatory dysregulation.af. or inflammatory disease.af. or inflammaging.af. or pro-inflammatory.af. or interleukin.af. or cardiometabolic diseases.af. or cardiometabolic markers.af. or chronic cardio metabolic.af. or chronic inflammation.af. or metabolic system.af. or metabolic response.af. or metabolic dysregulation.af. or metabolic biomarker.af. or metabolic disease.af. or metabolism.af. or renal function.af. or kidney function.af. [mp=title, abstract, heading word, drug trade name, original title, device manufacturer, drug manufacturer, device trade name, keyword, floating subheading word, candidate term word] 5247325

4 limit 3 to yr="1980 -Current" 4597318

5 (adult or adulthood or early adulthood or later life or life course or mid adulthood or older ages).af. 8720015

6 limit 5 to yr="1980 -Current" 8235412

7 2 and 4 383

8 6 and 7 139

**Supplementary Table 1**: Operationalization of Parental SEP Measures

| **SEP Measure** | **Coding Criteria (Low vs. High)** |
| --- | --- |
| Mother’s Education | Low: ≤ upper secondary; High: tertiary or above |
| Father’s Occupation | Low: manual/unskilled; High: professional/managerial |
| Parental Education | Low: both ≤ secondary; High: ≥ one tertiary |
| Parental Occupation | Low: both manual; High: ≥ one non-manual/professional |
| Family SEP | Low: bottom 2 quintiles; High: top 3 quintiles |
| Life course SEP | ﻿High: Parent graduated from HS/GED or higher  High: High financial level growing up |

**Supplementary Table 2. The characteristics of the included individual studies.**

| **n** | **Citation** | **Study design and data sources** | **Country** | **Follow-up and duration** | **Sample size (n)** | **Age range** | **Sex distribution** | **Ethnicity distribution** | **Exposures investigated** | **Biomarkers** | **Covariates** | **Effect direction/findings** | **Quality assessment** |
| --- | --- | --- | --- | --- | --- | --- | --- | --- | --- | --- | --- | --- | --- |
| 1 | Leino et al. (Leino et al., 1999) | Cohort study  Cardiovascular Risk in Young Finns Study | Finland | 1983, 1986, 1986, 1989 and 1992: 12-year follow-up | 536 | 21-30 | Females  93  Males 443 | Not reported | Parental education | Cholesterol, blood pressure, WC, triglycerides, BMI | All the analyses were gender specific | More maternal education linked to lower total/LDL cholesterol in daughters (-0.13/-0.12 mmol/L per category), but not sons. | Low risk |
| 2 | Karlamangla et al. (Karlamangla et al., 2005) | Cohort study, initiated in 1985.  Coronary Artery Risk Development in Young Adults (CARDIA) Study | United States | From 1985 to 1995: 10-year follow-up. | 4,149 | 18–30 | Females 2,258 Males 1891 | Not reported | Parental education | BMI blood pressure, fasting glucose, fasting insulin, waist–hip circumference ratio, LDL, HDL | Age, gender, race, lifestyle habits (smoking/alcohol/exercise), access to medical care. | - Inverse associations between indicators of SES and summary scores of baseline risk, risk change, and accumulated risk. - Parental education had the most consistent associations with risk scores in all race/gender groups. | Some concerns |
| 3 | Kivimäki et al. (Kivimäki, Smith, et al., 2006) | Cohort Study  Young Finns Study | Finland | 1983 and 2001 | 1,807 | 24-39 | Females 993  Nales  814 | Not reported | Parental education and parental occupation | Blood pressure | BMI, Sex | Parental SEP linked to higher BP from childhood through adulthood, beyond adult risk factors/BMI. | Some concerns |
| 4 | Kivimaki et al. (Kivimäki, Davey Smith, et al., 2006) | Prospective cohort study  The cardiovascular risk in young Finns study | Finland | 1980 and 2001: 21 years follow-up | 1,922 | 24–39 | Females  1,066 Males 856 | Not reported | Parental occupation | BMI, SBP, total cholesterol, HDL cholesterol, triglyceride, and plasma glucose concentrations | Health risks | - Lower childhood SEP was associated with a 20% increase in the odds of having a waist circumference. 102 cm in men and 88 cm in women (overall p = 0.05).  - Childhood SEP was not associated with LDL cholesterol, triglycerides, and BMI. | High-risk |
| 5 | Kivimäki et al. (Kivimäki et al., 2018) | Cross-sectional analysis. The Finnish national register | Finland | 1983 and 2001 | 2,270 | 24-39 | No reported | Not reported | Parental occupation and parental SEP | Blood pressure | Sex and Age | - Parental SEP had minor or no effect on SBP, which remained across birth cohorts and for both indicators of parental SES. | Low risk |
| 6 | Stea et al. (Stea et al., 2009) | Cross-sectional study  Norwegian National Guard | Norway | Not reported | 750 | 18-26 | Not reported | Did not state ethnicity distribution | Parental education | BMI and Total cholesterol | Age and maternal education | - Inverse association with high educational level compared with low educational level of the fathers. | High-risk |
| 7 | Hovi et al. (Hovi et al., 2010) | Cohort Study | Finland | 1978-2001 | 238 | 18-27 | Not reported | Not reported | Parental education | Blood pressure | Age and Sex | Parental education did not explain their higher blood pressure. | High-risk |
| 8 | Phillips et al. (Phillips et al., 2009) | Cross-sectional study  University of Pittsburgh Adult Health and Behavior (AHAB) project | United States | 2001-2005 | 811 | 30–54 | Females 417 Males 394 | Europ-Americans African Americans  17% African American | Parental occupation | C-reactive protein (CRP) | Parental absence, sex | - Childhood and adult socioeconomic standing are associated inversely with a circulating marker of inflammation in a relatively healthy, mid-life community sample. | High-risk |
| 9 | Lehman et al. (Lehman et al., 2009) | Cohort Study  Coronary Artery Risk Development in Young Adults Study (CARDIA) | United States | Not applicable to this study | 2,739 | 33-45 | Females 1,519 Males 1,220 | 473 African American men, 688 African American women, 747 White men, and 831 White women. | Parental education | Blood pressure | Sex | Parental education directly predicted SBP changes. Low SES increased cardiovascular risk, affecting BP directly and via parental SEP. African Americans showed higher baseline BP and steeper increases, though pathways were similar across race/gender. | High-risk |
| 10 | Packard et al. (Packard et al., 2011) | Cross-sectional analysis. Scottish Index of Multiple Deprivation (SIMD) | United Kingdom | Not applicable to this study | 666 | 35-64 | Not reported | Not reported | Father's occupation | Inflammatory outcomes - Serum IL-6 levels, CRP |  | - Adverse levels of biomarkers of ill health in adults appear to be influenced by the father's occupation and childhood home conditions. | Low risk |
| 11 | Nielsen et al. (2019) | Danish Registry of Childhood and Adolescent Diabetes (DanDiabKids) | Denmark | 2000 and 2013 | 4,079 | 18-21 | Not reported | NA | Maternal education | HbA1c | Adverse Childhood Experiences | Large differences in HbA1c across maternal education were found. | Low risk |
| 12 | Chao et al. (Chao et al., 2014) | Cross-sectional analysis. Data from an entrance health check-up survey at a university | Taiwan | 2014 | 4,552 | The mean age of the participants was 21.9 | Females 1,325 Males 2,145 | Not reported | Parental education and parental occupation | Blood pressure BMI, Waist circumference | Lifestyle and family history of diabetes and hypertension | - Lower parental SEP was associated with central obesity.  - Subjects with lower parental SES were found to be shorter and heavier than those with higher SES. | Some concerns |
| 13 | Janicki-Deverts et al. (Janicki-Deverts et al., 2012a) | Retrospective Cohort study  Coronary Artery Risk Development in Young Adults Study (CARDIA) | nited States | ﻿From 1985 to 2006: 20 years follow-up | 4,077 | 18 to 30 | Females  2,239 Males  1,838 | Black and white participants. | Parental education | Blood pressure | Age, race, alcohol consumption, physical activity, smoking. | - Higher father's and mother's educations were associated with a lower average baseline SBP and increase in SBP over time.  - Childhood socioeconomic status may influence women's health independent of their adult status. | High-risk |
| 14 | Hagger-Johnson et al (Hagger-Johnson et al., 2012) | Cross-sectional analysis  Lothian Birth Cohort Study, 1936 | United Kingdom | 2003-2006 | 1,091 | 67-71 | Females  543  Males 548 | Not reported | Father's occupation | C-reactive protein (CRP) and BMI | Sex and ethnicity | - Parental SES linked to inflammation via BMI and health behaviors, but not quality of life. | High-risk |
| 15 | Bennett et al. (Bennett et al., 2014) | Cross-sectional analysis  Jamaica 1986 Birth Cohort Study. | Jamaica | 2005 and 2007 | 746 | ﻿18–20 | ﻿Males  342 Females 404 | Afro-Caribbean | Parental education | High-sensitivity C-reactive protein (hs-CRP) | Sex ﻿ | - Parental education was associated with significantly higher odds of high hs-CRP. | Some concerns |
| 16 | Savitsky et al. (Savitsky et al., 2016) | Cohort study  The Jerusalem Perinatal Family Follow-Up Study | Israel | 1974 and 1976; 2003 and 2006 | 1,132 | 30–35 | Females  549  Males  583 | Not reported | Parental education and parental occupation | BMI, blood pressure, total cholesterol, HDL, triglyceride, plasma glucose | Gender, ethnic origin, and socioeconomic characteristics. | - Childhood-occupational SEP was negatively associated with body mass index, fat percentage, insulin, triglycerides, and low-density lipoprotein cholesterol, independent of adulthood SEP. | High-risk |
| 17 | Cabral et al (2019) | Cohort study | Portugal | 2003/2004, 2007-2008, 2011-2013 | 1147 participants | 18-21 | 52.0% females | NA | Parental education | High sensitivity C reactive protein  A, BMI WC | Physical activity and diets | A significant longitudinal effect of the accumulation of adiposity on low-grade inflammation was observed. | Some concerns |
| 18 | John-Henderson et al. (John-Henderson et al., 2016) | Cohort Study  Phase II of the Adult Health and Behavior project. | United States | Not applicable to this study | 457 | mean age = 42.75 years | Females  242  Males  215 | 80% white, | Parental education | Serum IL-6 levels | Sex | - Childhood SES has a marginally significant main effect on IL-6, which seems to be moderated by recent life events. Low childhood SES exhibit an inflammatory phenotype in the context of a high number of recent negative life events. | Low risk |
| 19 | Al Hazzouri et al. (Zeki Al Hazzouri et al., 2015) | Cross-sectional study  Sacramento Area Latino Study on Aging | United States | 1998-1999 | 1,789 | 60-101 | Not reported | US-born or 51.1% foreign-born (in Mexico or another Central or South American country) | Parental education | Waist circumference | Time since migration and hypertension | - US-born adults with high education had 37% lower diabetes odds than those with low education, regardless of parental education. Foreign-born adults needed both high parental and personal education for 55% lower odds of large waist circumference. | Some concerns |
| 20 | Friedman et al. (Friedman et al., 2015) | Cohort Study. National Survey of Midlife Development in the United States (MIDUS) | United States | Second wave | 1,180 | ﻿25-74 | Not reported | white and non-white races | Parental education and parental occupation | Allostatic load | Race, age, and Sex. | - Childhood SEP and physical abuse were associated with increased AL.  - For each adverse experience in childhood, AL in middle life increased by 0.093. | Low risk |
| 21 | Boylan et al. (Boylan et al., 2020) | Cohort study | United States | ﻿1987–1988  2 years follow-up | 246 | 30–34 | Not reported | Black and White | Parental education and parental occupation | Blood pressure | Age ﻿ | Lower childhood SES predicted higher HR/SBP recovery, independent of covariates. Men with fewer psychological resources showed stronger SBP effects. | High-risk |
| 22 | Barboza-Solís et al (Barboza Solís et al., 2016) | Cohort study since 1958  British Registrar General's social class system (RGSC) | United Kingdom | ﻿Cohort members between 7y and 50y: 60 years follow up | ﻿7,573 | 44 | Females  3,791 Males  3,782 | Not reported | Mother education | Allostatic load | Sex ﻿ | Lower maternal education and manual paternal occupation predicted higher allostatic load at age 44, mediated by educational, financial, and health behavior pathways in both genders. | Low risk |
| 23 | Doom et al. (Doom et al., 2017) | Cohort study  National Longitudinal Study of Adolescent to Adult Health | United States | Not reported | 14,493 | 24-34 | Not reported | White, black/African American, Hispanic | Parental occupation and parental SEP | BMI, blood pressure, fasting & non-fasting glucose | Age, sex, BMI, smoking, SBP, diabetes, medication use, ﻿self-reported health. | Higher SES linked to 10% lower CVD risk; childhood adversity raised risk by 6%. Both significantly impacted young adult CVD. | Low risk |
| 24 | Christensen et al. (Dinne S. Christensen et al., 2018) | Cohort Study  Copenhagen Perinatal Cohort | Denmark | 1982–1994 2009–2011 | 668 | 27 - 50 | Females 361  Males  307 | Not reported | Parental occupation, education, parental SEP | Allostatic load | Maternal smoking, BMI, complications at birth | - Parental socioeconomic position at one year was inversely associated with midlife allostatic load. Results suggest that part of this association was mediated by education. | Low risk |
| 25 | Christensen et al. (Dinne Skjærlund Christensen et al., 2018) | Cohort Study  Copenhagen Perinatal Cohort | Denmark | 1959-1961 and 2009-2011 | 1648 | 49–52 | Females  922  Males  726 | Not reported | Parental occupation, education, and parental SEP | Allostatic load | Age, sex. | - Parental SEP at one year was the only significant predictor in the social model, with lower levels of SEP related to higher AL scores. | High-risk |
| 26 | Präg and Richards (Präg & Richards, 2019) | Cross-sectional analysis. UK Household Longitudinal Study (UKHLS) | United Kingdom | 2010–2012 2011–2012 | 9,851 | 52 | Not reported | White participants | Parental occupation | Allostatic load | Age, Sex, ethnicity, partnership status and labour market status. | Working class shows highest allostatic load, salariat lowest, with intermediate classes between | Low risk |
| 27 | Berger et al. (Berger et al., 2019) | Cohort Study. Skipogh, CoLaus, Whitehall and ELSA | Europe | 1958 and 2013 | 13,078 | 44-76 | Females  5,232  Males  7,846 | Not reported | Father's occupation | C-reactive protein (CRP) | Alcohol consumption, smoking, and sedentary lifestyle. | Lower parental SEP linked to higher CRP, but attenuated after adjusting for education/occupation; remained significant for childhood SEP in both sexes. | Low risk |
| 28 | Lunyera et al. (Lunyera et al., 2020) | Prospective cohort study. Jackson Heart Study | United States | 2000–2004; 2005–2008; 2009–2013 | 3421 (63% female) | 21–94 | Not reported | Not reported | Parental education | CRP, SBP, metabolic outcomes, and allostatic load | Age, sex, smoking, use of routine care, CVD. | Lower lifetime SES linked to higher allostatic load and increased CKD risk. | Low risk |
| 29 | Kjøllesdal et al (Kjøllesdal et al., 2017) | Cohort Study    Norwegian health surveys | Norway | 1974–2003 | 271,643 | 40–44 | Females 139,473 Males 132,170 | Not reported | Parental occupation and parental SEP | Blood pressure, Total Cholesterol, BMI | Sex | - Early life family factors are associated with risk factors and mortality from CVD and IHD | Some concerns |
| 30 | Hsuan Lin et al. (Lin et al., 2017) | Cohort study | Taiwan | 1999, 2000, 2006 | 1,036 | ≥60 years | Females 479  Males  557 | Asian and non-Asian populations | Father's education and occupation | C reactive protein (CRP) and Interleukin (IL-6) | Sex and ethnicity | - Positive association. Life-course SEP predicts inflammatory markers in older age. Low SEP in childhood is related to elevated inflammatory markers in older age | Low risk |
| 31 | Chen et al. (Chen et al., 2012) | Cohort study | United States | 1995-1996  2004-2006 | 1,207 | 25-74 | Females 681  Males 526 | White and non-white population | Parent's higher educational attainment | Allostatic load | Sex and ethnicity | - Positive association. Adults from low-childhood SES backgrounds had higher allostatic load scores than adults from high-childhood SES backgrounds. | Low risk |
| 32 | Tabassum et al. (Tabassum et al., 2008) | Cohort study | United Kingdom | 1958 | 5,951 | 45 | Females 2,817  Males 3,134 | 97% white population | Parental occupation | C-reactive protein (CRP) | Age and Sex | - Positive association. Risk exposure related to SEP accumulates across the life course and contributes to raised levels of C-reactive protein. | Low risk |
| 33 | Wannamethee et al. (Wannamethee et al., 1996) | Cross-sectional study  British Regional Heart Study | United Kingdom | 1978 and 1980 | 5,934 | 40-59 | Males |  | Father's occupation | SBP, total cholesterol, HDL, blood glucose | Age | - Positive association. father's manual occupation influenced ischemic heart disease risk in adult life | Low risk |
| 34 | Boylan et al. (Boylan et al., 2016) | Cohort Study. Midlife in the United States (MIDUS) | United States | NA | 1,578 | 25-85 | Not reported | Black and white participants | Parental occupation, education, and parental SEP | Inflammatory outcomes - Serum IL-6 levels, CRP | Age, Sex, race | Black adults with lower childhood SES had higher CRP (R²<.01); IL-6 showed similar but weaker associations. | Low risk |
| 35 | Prior 2021 | British Household Panel Survey and Understanding Society | United Kingdom | Collected between 2011 and 2012 | 3210 individuals | Mean 51 years | 54% females | NA | Deprivation exposure history | Allostatic Load index is constructed from 13 biomarkers | Age, sex, education, employment, tenure, marital status | There is a gradient in allostatic load by histories of deprivation exposure. | Low risk |
| 36 | Gustafsson et al. (2011) | Cohort study - ﻿The Northern Swedish Cohort | Sweden | ﻿1983, 1986, 1995 and 2008 | 855 | 43 years | ﻿391 women and 417 men | NA | Life course SES and parental occupation status | Allostatic load using 12 biomarkers | Health behaviours | SES over the life course influences the level of multi-systemic dysregulation in mid-adulthood, with the strongest support for the cumulative risk model. | Low risk |
| 37 | Surachman et al. 2020 | The Midlife in the United States Refresher study. | United States | ﻿1995 to 1996, followed by the second wave in 2004 and 2011 | 863 | Mean 52 years | 281 female (47%) | ﻿592 non-Hispanic white and 158 non-Hispanic black participants | Life course SES (education, occupation, income) | Inflammation (CRP and IL-6) | Education, social mobility | The lack of SES mobility differentiation on inflammation is an indication of diminished return for the most affluent class among black participants. | Low risk |
| 38 | Castagné et al. (2016) | Cohort Study – European Prospective Investigation into Cancer and Nutrition (EPIC-Italy) | Italy | Not reported | 268 | Mean 53 years | 170 females | NA | Life course SES and father’s occupational position | Inflammation and BMI | Disease and smoking status | ﻿ Early life SES impacts adults physiology | Low risk |

**Supplementary Figure 1. Funnel plots.**

Funnel plot. Blood pressure (cohort studies)

Number of studies: k = 12


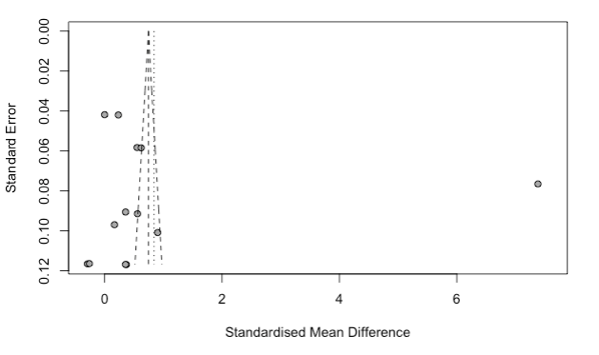


Funnel plot. Blood pressure (cross sectional)

Number of studies: k = 7


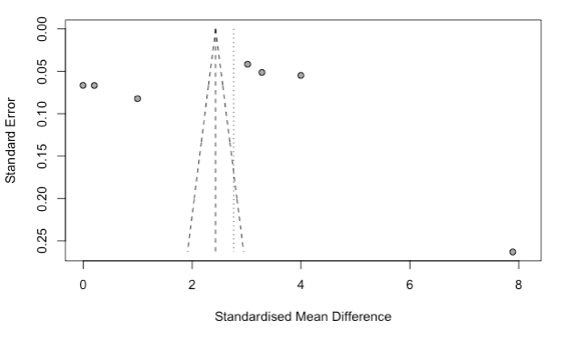


Funnel plot. Glucose metabolism (cohorts)

Number of studies: k = 5


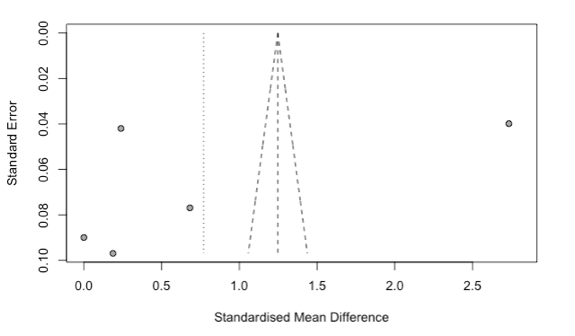


Funnel plot. Lipid metabolism (cohorts)

Number of studies: k = 13


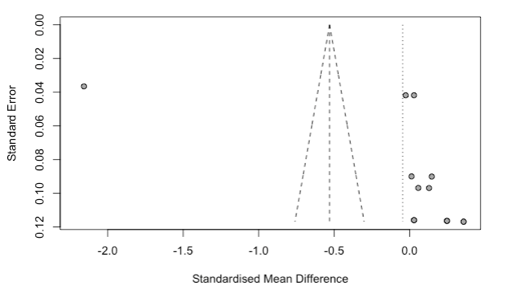


Funnel plot. Adiposity (cohorts)

Number of studies: k = 12


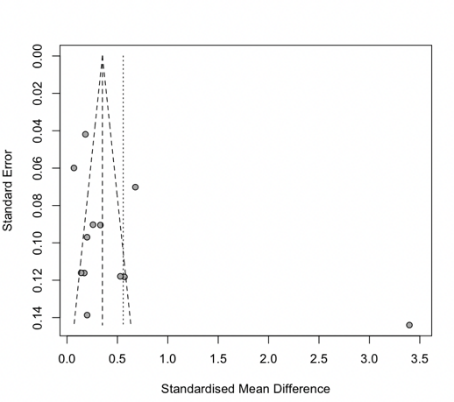


Funnel plot. CRP (cohorts)

Number of studies: k = 11


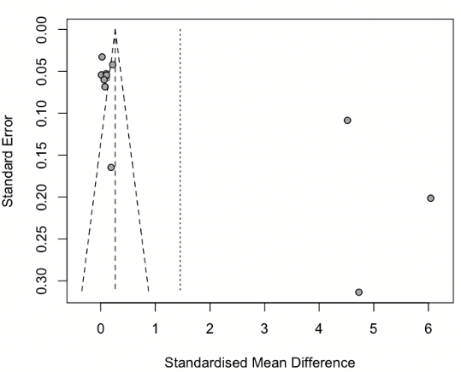


Funnel plot. IL-6 (cohorts)

Number of studies: k = 5


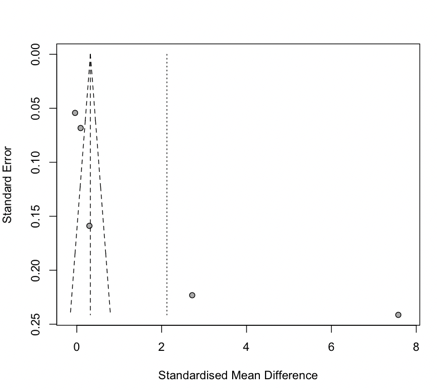


Funnel plot. CRP and IL-6 (cross sectional)

Number of studies: k = 5


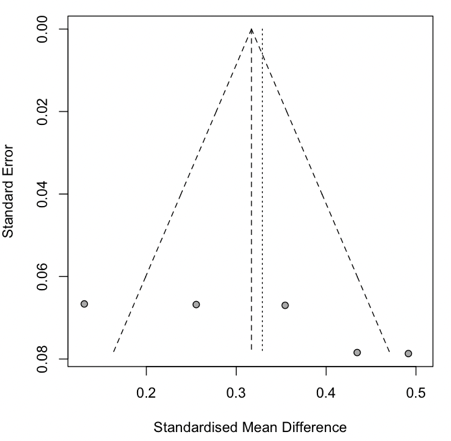

Supplement: Supplementary file 1 — Data S1. [file ECI-56-e70125-s001.docx]
